# Protective effects of dexmedetomidine on cerebral ischemia/reperfusion injury via the microRNA-214/ROCK1/NF-κB axis

**DOI:** 10.1186/s12871-021-01423-5

**Published:** 2021-08-16

**Authors:** Wenyi Liu, Cuihua Shao, Chuanshan Zang, Jian Sun, Min Xu, Yuna Wang

**Affiliations:** 1 Department of Anesthesiology|, The Affiliated Hospital of Qingdao University, No. 59, Haier Road, Laoshan District, Qingdao, 266003 Shandong PR China; 2 Department of Obstetrics, The Affiliated Hospital of Qingdao University, Qingdao, 266003 Shandong PR China; 3 Department of Otorhinolaryngology Head and Neck Surgery, The Affiliated Hospital of Qingdao University, Qingdao, 266003 Shandong PR China; 4 Department of Orthopaedics, The Affiliated Hospital of Qingdao University, Qingdao, 266003 Shandong PR China

**Keywords:** Cerebral ischemia/reperfusion injury, Dexmedetomidine, microRNA-214, Rho-associated kinase 1, NF-κB

## Abstract

**Background:**

Cerebral ischemia/reperfusion injury (CIRI) is a complication of surgical procedure associated with high mortality. The protective effect of dexmedetomidine (DEX) on CIRI has been explored in previous works, yet the underlying molecular mechanism remains unclear. Our study explored the protective effect of DEX and its regulatory mechanism on CIRI.

**Methods:**

A CIRI rat model was established using middle cerebral artery occlusion (MCAO). Neurological deficit scores for rats received MCAO modeling or DEX treatment were measured. Cerebral infarction area of rats was detected by TTC staining, while damage of neurons in hippocampal regions of rats was determined by hematoxylin-eosin (HE) staining. Apoptosis rate of neurons in hippocampal regions was examined by TUNEL staining. The dual-luciferase assay was performed to detect the binding of microRNA-214 (miR-214) to Rho-associated kinase 1 (ROCK1).

**Results:**

DEX treatment significantly reduced infarction area of MCAO rats and elevated miR-214 expression. Injection of miR-214 inhibitor attenuated the effect of DEX in MCAO rats by increasing the area of cerebral infarction in rats and apoptosis rate of hippocampal neurons. ROCK1 was targeted and negatively regulated by miR-214. The overexpression of ROCK1 led to activation of NF-κB to aggravate CIRI.

**Conclusion:**

Therapeutic effects of DEX on CIRI was elicited by overexpressing miR-214 and impairing ROCK1 expression and NF-κB activation. Our finding might provide novel insights into the molecular mechanism of DEX in rats with CIRI.

**Supplementary Information:**

The online version contains supplementary material available at 10.1186/s12871-021-01423-5.

## Background

Cerebral ischemia/reperfusion injury (CIRI) is often induced by ischemic stroke which is caused by arterial occlusion, leading to long-term disability and even death [1]. CIRI is also a devastating complication of neurological and cardiovascular surgeries [2]. Moreover, the neurodegenerative disorders caused by CIRI significantly impair the memory and learning ability, limb use and other neurological performances [3]. Although the mortality caused by CIRI is largely reduced, the incidence of accompanied ischemic stroke remains high [4]. Therefore, more potential therapeutic options for CIRI need to be studied.

Previous clinical evidence has proposed that dexmedetomidine (DEX) could enhance the cardiac and neurological surgeries outcomes and relieve the pain of sufferers [5]. DEX is a kind of α2-adrenergicreceptor agonist that possesses analgesic and sedative properties [6]. Moreover, DEX has already been reported to exert protective effects against IRI of various organs, including the heart and the kidney and to be neuroprotective against CIRI in rats, yet the underlying mechanism remains to be elucidated [7]. In recent works, microRNAs (miRNAs) have been indicated to be involved in the neuroprotective effects of DEX. For instance, miR-340 could enhance the therapeutic impacts of DEX on neuroinflammation [8]. Similarly, miR-128 strengthens neuroprotective effects of DEX on neonatal mice with CIRI [9]. Interestingly, miR-214 participates in the regulation of CIRI in rats with unspecified molecular mechanism [10]. However, limited studies investigated the involvement of miR-214 in DEX treatment. Rho-associated kinase 1 (ROCK1) has been identified as a target gene of miR-214 in osteosarcoma cells [11]. Nevertheless, the relation between miR-214 and ROCK1 has rarely been reported in CIRI. ROCK1, a member of the AGC kinases family and a significant mediator of mammalian cell motility via the regulation of cytoskeleton [12] has also been reported to regulate the neuronal apoptosis induced by CIRI [13]. Furthermore, ROCK1 could promote the phosphorylation of nuclear factor kappa-light-chain-enhancer of activated B cell (NF-κB) by activating TLR4, thereby promoting the development of inflammation in cornea cells [14]. NF-κB is extensively investigated in CIRI, and impairment of the NF-κB pathway may provide a therapeutic strategy for CIRI [15, 16]. In the present study, we postulated that miR-214, ROCK1, and NF-κB may be involved in DEX-mediated protective effects against CIRI in rats. Therefore, this study was conducted to validate our assumption and to investigate the impacts of DEX-regulated miR-214 as well as the relevant regulatory mechanism on CIRI using Sprague Dawley (SD) rats with middle cerebral artery occlusion (MCAO).

## Methods

### Animal experiments

A total of 100 healthy specific-pathogen-free SD adult male rats (aged 8–10 weeks; weight 200–250 g) were purchased from Beijing Vital River Laboratory Animal Technology Co., Ltd. (Beijing, China) (97 rats were actually used and the remaining three were used for other studies). Rats were acclimatized to the laboratory for 1 week before experiments, during which they had a free access to feed and water. Room temperature was set at 22 ± 2 °C with a relative humidity at 50–60% and 12:12 h light-dark cycle. Ventilation was performed regularly. Mats were replaced to keep rats healthy. The sample size of the animals and the flow chart of the study are shown in Supplementary Material. The animals were divided into 8 groups. Establishment of the model was repeated as necessary to ensure that each group had the required number of animals (*n* = 10) (Table 1). The mortality rates of rats for each MCAO-based experiments are exhibited in Table 2.

**Table 1:** Grouping for experimental animals

Group (*n* = 10)	Surgerical procedures
sham	Procedures for anesthesia were the same as that for the MCAO group, except for the occlusion of middle cerebral artery
DEX	Based on the sham group, DEX was intravenously administered at a loading dose of 1 μg/kg at the very beginning of the surgery, and was then administered at 0.05 μg/kg/min for the next two hours
MCAO	MCAO modeling
MCAO + DEX	Simultaneous treatment of MCAO modeling and DEX
NC inhibitor/miR-214 inhibitor	Based on the operation of MCAO + DEX, NC inhibitor/miR-214 inhibitor (80 nM) with invivofectamine was administered via intracerebroventricular infusion half an hour before surgery
oe-NC/oe-ROCK1	Based on the operation of MCAO + DEX, oe-NC/oe-ROCK1 (100 nM) with invivofectamine was administered via intracerebroventricular infusion half an hour before surgery

Plasmids of miR-214 inhibitor, oe-ROCK1 and the matched NC were purchased from GenePharma (Shanghai, China)

*Notes*: *DEX* Dexmedetomidine, *MCAO* Middle cerebral artery occlusion, *ROCK1* Rho-associated kinase 1, *miR-214* MicroRNA-214, *NC* Negative control

**Table 2:** Animal mortality rates for each MCAO-based experiments

Group	Mortality rates (based on 10 rat/per group)
MCAO	1/10 (10%)
MCAO + DEX	2/10 (20%)
NC inhibitor	3/10 (30%)
miR-214 inhibitor	2/10 (20%)
oe-NC	1/10 (10%)
oe-ROCK1	3/10 (30%)

*Note*: *MCAO* Middle cerebral artery occlusion, *DEX* Dexmedetomidine, *miR-214* MicroRNA-214, *NC* Negative control, *oe* Overexpression

### MCAO modeling and neurological function evaluation

The rats were fasted for 12 h before surgery, yet having a free access to water. The MCAO model was established referring to Zea-Longa method, followed by the neurological function evaluation after 24 h [17]. The rats were anesthetized by an intraperitoneal injection of 10% chloral hydrate solution (300 mg/kg) and fixed in a supine position. The internal and external carotid arteries of the common carotid were carefully separated, whilst proximal common end of the common carotid artery and the distal end of the external carotid artery were ligated. A nylon threaded bolt was slowly inserted into the internal carotid artery and secured with a retaining wire. After occlusion of blood flow for 2 h, the bolt was pulled out, followed by a 24-h reperfusion. Eventually, the wound was sutured layer by layer, during which the ambient temperature was maintained at 37 ± 0.5 °C with rectal temperature, respiratory rate and heart rate of rats monitored. The awakened rats were put back to the room for further observation.

Twenty-four h after operation, the neurological function of each rat was evaluated by scoring: 0 point for rats without neurological symptoms; 1 point for rats that could not fully extend the contralateral forepaw when tails were raised (indicating a mild neurological deficit); 2 points for rats turned to the other side of the operation while walking (indicating a moderate neurological deficit); 3 points for rats fell to the left (indicating a severe focal deficit); 4 points for rats that could not walk on their own or lose consciousness. Rats with neurological deficit scores ranging from 2 to 3 were taken as successful modeled MCAO rats. Rats not conforming to the criteria and those experienced subarachnoid hemorrhage or died within 24 h were excluded. Other rats were randomly selected and received experimental procedures. A total of 17 MCAO modeled rats did not meet the requirements. At the end of the experiment, all alive rats were euthanized by intraperitoneal injection of sodium pentobarbital at 200 mg/kg.

### 2, 3, 5-Triphenyltetrazolium chloride (TTC) staining

Five rats from each group were euthanized by an intraperitoneal injection of sodium pentobarbital (200 mg/kg). The brain tissues were harvested, paraffin-embedded, and cut into 2-mm thick coronal sections. The sections were dewaxed by xylene, dehydrated by gradient ethanol, stained with 10 g/L TTC solution (Solarbio, Beijing, China) for 15 min, and fixed with 4% paraformaldehyde. Normal brain tissues were stained in red, whereas infarcted tissues in white. The infarction area was calculated by ImageJ.

### Hematoxylin-eosin (HE) staining

The remaining five rats in each group were euthanized by an intraperitoneal injection of 200 mg/kg sodium pentobarbital. The isolated hippocampal tissues were fixed in 4% paraformaldehyde solution, paraffin-embedded, and sectioned (thickness of 5 μm) with a paraffin slicer (Leica, Wetzlar, Germany). After being dewaxed by xylene and dehydrated by gradient ethanol, hippocampal tissue sections were stained with hematoxylin (Sigma-Aldrich, St. Louis, MO, USA) for 5 min and differentiated with ethanol hydrochloride for 30 s. A 2-min eosin staining (Sigma-Aldrich) was then performed. After routine dehydration, clearing, and mounting, hippocampal neurons were observed under a 400-fold optical microscope (Olympus BX51, Olympus, Tokyo, Japan).

### Terminal deoxynucleotidyl transferase-mediated dUTP-biotin nick end labeling (TUNEL)

Paraffin-embedded rat hippocampal tissues were sectioned (thickness of 5 μm), dewaxed and dehydrated. Apoptotic neuronal cells were quantified by a TUNEL apoptosis detection kit (ZSJQ Biotechnology, Beijing, China) and observed under the light microscopy (BX50; Olympus) in five randomly selected fields. Normal nuclei were stained in blue, while positive apoptotic cells in brown-yellow. TUNEL-positive cells were measured by ImageJ.

### Microarray analyses

Variation of miRNAs in paraffin-embedded brain tissues of rats in the MCAO group and the MCAO + DEX group (*n* = 3) was analyzed by SurePrint Rat miRNA Microarrays (Agilent, Santa Clara, CA, USA). Data were retrieved and analyzed by Agilent feature extraction software, and raw data were normalized using quantile normalization. Other analyses were conducted through GeneSpring GX software (Agilent).

### Reverse transcription quantitative polymerase chain reaction (RT-qPCR)

Total RNA was extracted by RNAiso Plus (TaKaRa, Tokyo, Japan). Reverse transcription was conducted by reverse transcription reagents (TaKaRa), and amplification by SYBR Green Master Mix (TaKaRa) in Light Cycler 480II (Roche Diagnostics, Co., Ltd., Rotkreuz, Switzerland). U6 or glyceraldehyde-3-phosphate dehydrogenase (GAPDH) served as loading controls. Primers used in this experiment are shown in Table 3.

**Table 3:** Primer sequences for RT-qPCR

Targets	Sequences (5′-3′)
miR-214	F: AGAGTTGTCATGTGTCT
	R: GAACATGTCTGCGTATCTC
ROCK1	F: CACGCCTAACTGACAAGCACCA
	R: CAGGTCAACATCTAGCATGGAAC
SOX4	F: GATCTCCAAGCGGCTAGGCAAA
	R: GATCTCCAAGCGGCTAGGCAAA
SEMA4C	F: GGAGTATGACTGCTATTCCGAGC
	R: ACACCAACCGAGCCTTCAGGAA
PPTC7	F: GCGGTTAGTGAAAGAAGGACGC
	R: TTCTGTCCAGCACCACGATGCA
U6	F: CTCGCTTCGGCAGCACAT
	R: TTTGCGTGTCATCCTTGCG
GAPDH	F: CATCACTGCCACCCAGAAGACTG
	R: ATGCCAGTGAGCTTCCCGTTCAG

*Notes*: *RT-qPCR* Reverse transcription quantitative polymerase chain reaction, *F* Forward, *R* Reverse, *miR-214* microRNA-214, *ROCK1* Rho-associated kinase 1, *SOX4* SRY-box transcription factor 4, *SEMA4C* Semaphorin 4C, *PPTC7* Protein phosphatase targeting COQ7, *GAPDH* Glyceraldehyde-3-phosphate dehydrogenase

### Dual-luciferase reporter assay

The putative binding sequence of miR-214 in ROCK1 3′-untranslated region (UTR) was obtained through Starbase (http://starbase.sysu.edu.cn/), based on which mutation of the binding site was designed. The sequence was cloned to the downstream of luciferase gene in the pmirGLO luciferase vector (Promega, Madison, WI, USA) to generate the luciferase reporter plasmids ROCK1-wild type (WT)/ROCK1-mutant type (MT), which were co-transfected with miR-214 mimic or negative control (NC). Relative luciferase activity was measured with a dual-luciferase reporter assay system (Promega).

### Immunohistochemistry

Briefly, paraffin-embedded rat hippocampal tissue sections (thickness of 5 μm) were deparaffined, hydrated, and treated with 3% H_2_O_2_ for 10 min to block endogenous peroxidase activity. Non-specific binding was offset by 5% bovine serum albumin (BSA). Next, the sections were incubated with primary antibodies to ROCK1 (1:100, ab134181, Abcam, Cambridge, UK) or phosphorylated NF-κB p65 (phospho-S529) (1:100, ab97726, Abcam) for 2 h at room temperature, and with secondary goat anti-rabbit IgG H&L (horseradish peroxide, 1:2000, ab205718, Abcam) for 30 min, followed by another a 30-min incubation with streptavidin-horseradish peroxidase complex. The sections were then stained by diaminobenzidine, counterstained with hematoxylin, fixed, and observed under a microscope with 4 visual fields randomly selected. The positive rate was measured by ImageJ.

### In situ hybridization (ISH)

Paraffin-embedded rat hippocampal tissue sections (5 μm) were heated in a 60 °C oven for 2 h, dewaxed, and hydrated. The sections were treated with Proteinase K working solution at 37 °C for 5 min. The sections were incubated with primary antibody to NeuN (1:100, ab177487, Abcam) for 2 h at room temperature and then incubated with goat anti-rabbit secondary antibody to IgG H&L (Alexa Fluor® 488, 1:200, ab150077, Abcam) for 30 min at room temperature. A specific RNA hybridization probe for Cy5-labeled miR-214 (Abologist, Shanghai, China) was subsequently added for a 1-h incubation at 55 °C, followed by a 3-h hybridization at 37 °C. The nuclei were stained and sealed using 4′,6-Diamidino-2-Phenylindole staining and sealing agent (Cell Signaling Technologies, Beverly, MA, USA). Finally, the expression of miR-214 (red) in neuronal regions of rat hippocampal tissues (NeuN labeled, green) was observed under a fluorescence microscopy (Olympus), and ImageJ was used for quantitative analysis.

### Statistics

All quantitative data conform to normal distribution were exhibited as mean ± standard deviation. Three independent experiments were carried out. Statistical analysis was performed using SPSS 22.0 software (SPSS, Inc. Armonk, NY, USA). Data between two groups were compared using unpaired *t* test, data among multiple groups using two-way or one-way analysis of variance (ANOVA) with Tukey’s post-hoc test. *p* < 0.05 represents statistically significant.

## Results

### DEX ameliorates CIRI in MCAO rats

**Fig. 1:**
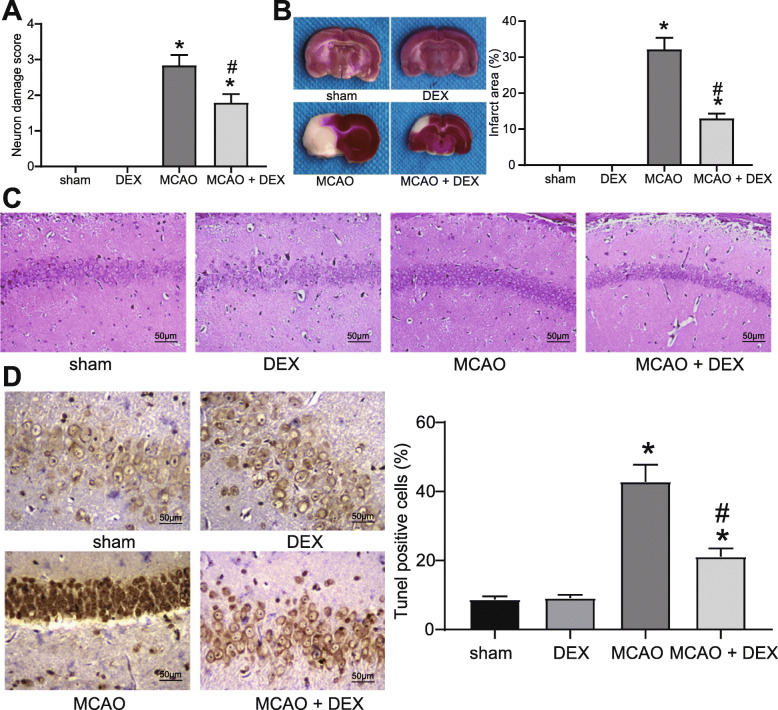
DEX ameliorates CIRI in rats. **A** Neurological deficit scores for rats in each group. **B** Cerebral infarction area of rats detected by TTC staining. **C** Damage of neurons in hippocampal regions determined by HE staining. **D** Apoptosis rate of neuronal cells in hippocampal regions examined by TUNEL staining. For panel A, B, and D, comparisons were made using one-way ANOVA. * *p* < 0.05 compared with the sham group; # *p* < 0.05 compared with the MCAO group

To explore the therapeutic effects of DEX on rats with CIRI, we scored the neurological function of rats at 24 h post-MCAO (Fig. 1A). There was no significant change of the neurological deficit score between the DEX group and the sham group, suggesting that treatment of DEX alone did not affect neurotoxicity in rats. However, neurological deficit scores for rats in the MCAO and the MCAO + DEX groups were higher than those in the sham group, yet the MCAO + DEX group showed reduced neurological deficit scores relative to the MCAO group.

Next, TTC staining was performed on coronal sections of rats, which showed that the area of cerebral infarction increased in rats with CIRI, and DEX partially reduced infarction area (Fig. 1B). Subsequently, the neurological damage in the hippocampal tissues was detected (Fig. 1C, D). HE staining revealed that neurons in the hippocampal CA1 region of rats in the sham and the DEX groups were regularly aligned, which exhibited intact cellular structure with round, large, and clearly visible nuclei. In contrast, MCAO rats showed obvious neuronal damage with irregularly shaped cells, concentrated cytoplasm and nuclei, and impaired hippocampal structure. The neuronal damage of the MCAO + DEX group was ameliorated versus the MCAO group.

### DEX induces miR-214 expression in rats with CIRI

**Fig. 2:**
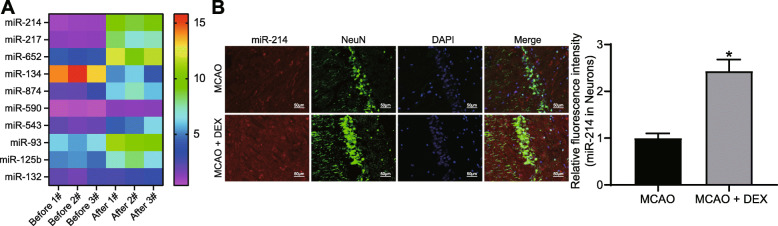
Differentially expressed miRNAs in DEX-treated rats underwent MCAO. **A** The differentially expressed miRNAs in the MCAO rats with or without DEX treatment screened using microarray analysis (*n* = 3). **B** Detection of miR-214 expression in rat neurons (green) by ISH combined with immunofluorescence staining. For panel B, comparisons were made using unpaired *t* test. * *p* < 0.05 compared with the MCAO group

To understand the mechanism of DEX affecting CIRI, microarray analysis of brain tissues in the MCAO rats with or without DEX treatment was conducted to screen out differentially expressed miRNAs induced by DEX treatment. The top ten differentially expressed miRNAs are shown in Fig. 2A. Among them, miR-214 showed the most remarkable difference after DEX treatment in brain tissues of MCAO rats. The effect of DEX on miR-214 expression in rat hippocampal neurons was detected by ISH combined with immunofluorescence assay. We observed significantly elevated levels of miR-214 (red) in NeuN-labeled (green) hippocampal neurons of DEX-treated MCAO rats (Fig. 2B).

### Inhibition of miR-214 expression suppresses the ameliorating effects of DEX on CIRI

**Fig. 3:**
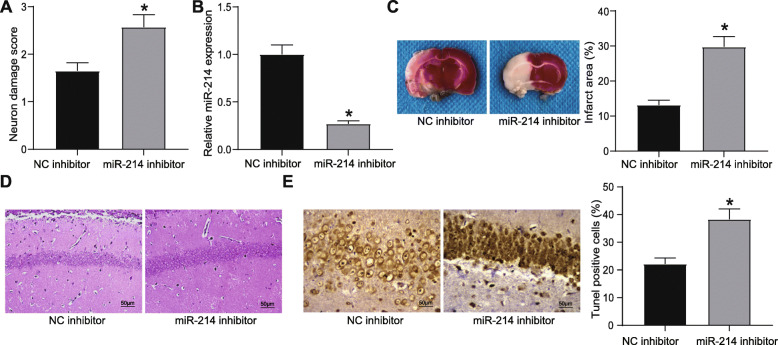
DEX attenuates CIRI in rats with MCAO by increasing miR-214 expression. **A** Neurological deficit scores for rats injected with miR-214 inhibitor. **B** miR-214 expression in rat brain tissues detected by RT-qPCR. **C** Infarction area of rats injected with miR-214 inhibitor observed by TTC staining. **D** Damage of neurons in hippocampal regions determined by HE staining. **E** Apoptosis rate of neurons in hippocampal regions examined by TUNEL staining. For panel A, B, C, and E, comparisons were made using unpaired *t* test. * *p* < 0.05 compared with rats injected with NC inhibitor

To validate whether DEX ameliorated CIRI by upregulating miR-214, a rescue experiment was conducted. Rats were intraventricularly injected with miR-214 inhibitor and NC inhibitor half an hour before MCAO operation. After 24 h, the neurological deficit score for rats injected with miR-214 inhibitor was increased compared with that in the rats injected with NC inhibitor (Fig. 3A). RT-qPCR results displayed that miR-214 was downregulated in the brain tissues of rats injected with miR-214 inhibitor (Fig. 3B). TTC staining showed that the injection of miR-214 inhibitor increased the area of cerebral infarction in rats (Fig. 3C). Moreover, HE staining results suggested that injection of miR-214 inhibitor attenuated the repairing effect of DEX on CIRI in MCAO rats, as evidenced by changed morphology of neurons in rats (Fig. 3D). Apoptosis rate of hippocampal neurons was elevated by injection of miR-214 inhibitor, as TUNEL staining unraveled (Fig. 3E).

### miR-214 targets ROCK1

**Fig. 4:**
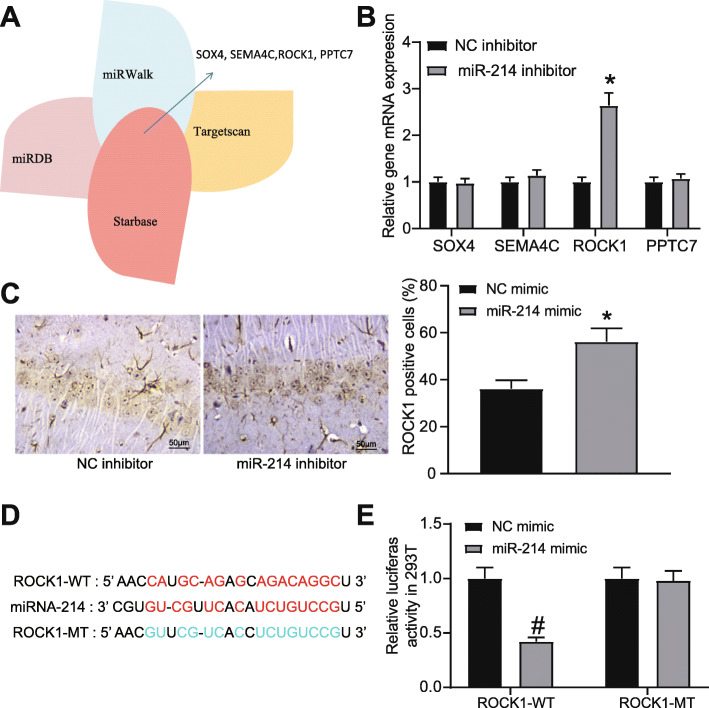
ROCK1 is the downstream target gene of miR-214. **A** Target genes of miR-214 predicted by Starbase, TargetScan, miRWalk and miRDB databases. **B** The mRNA expression of predicted target genes in brain tissues of rats injected with miR-214 inhibitor or NC inhibitor detected by RT-qPCR. **C** ROCK1 protein expression in brain tissues of rats injected with miR-214 inhibitor or NC inhibitor determined by immunohistochemistry. **D** Sequences for binding sites between miR-214 and ROCK1. **E** Luciferase activity of ROCK1-WT and ROCK1-MT after treatment of miR-214 mimic examined by dual-luciferase reporter assay. For panel C, comparison was made using unpaired *t* test. * *p* < 0.05 compared with rats injected with NC inhibitor. For panel B and E, comparisons were made using one-way or two-way ANOVA, respectively. * *p* < 0.05 compared with rats injected with NC inhibitor; # *p* < 0.05 compared with rats injected with NC mimic

To explore the downstream target of miR-214 in CIRI, the potential downstream target genes of miR-214 were predicted in Starbase, TargetScan, miRWalk and miRDB databases (Fig. 4A). The expression of the target genes in the intersection in the brain tissues of rats injected with NC inhibitor or miR-214 inhibitor was detected by RT-qPCR, which revealed that ROCK1 was the differentially expressed one (Fig. 4B). ROCK1 expression in the hippocampus of rats injected with NC inhibitor or miR-214 inhibitor was detected by immunohistochemistry, which showed that inhibition of miR-214 expression led to an increase of ROCK1 protein expression (Fig. 4C). Then, the potential binding sites between ROCK1 and miR-214 were obtained, based on which the mutation sequences were designed (Fig. 4D). After the sequences were inserted into the luciferase reporter plasmids ROCK1-WT and ROCK1-MT, the plasmids were co-transfected with miR-214 mimic into 293 T cells. At 48 h post co-transfection, the dual-luciferase reporter assay results showed that overexpressed miR-214 distinctly suppressed the luciferase activity of ROCK1-WT, but had no significant effect on the luciferase activity of ROCK-MT (Fig. 4E).

### Overexpressed ROCK1 dampens the therapeutic effects of DEX on CIRI through activation of the NF-κB pathway

**Fig. 5:**
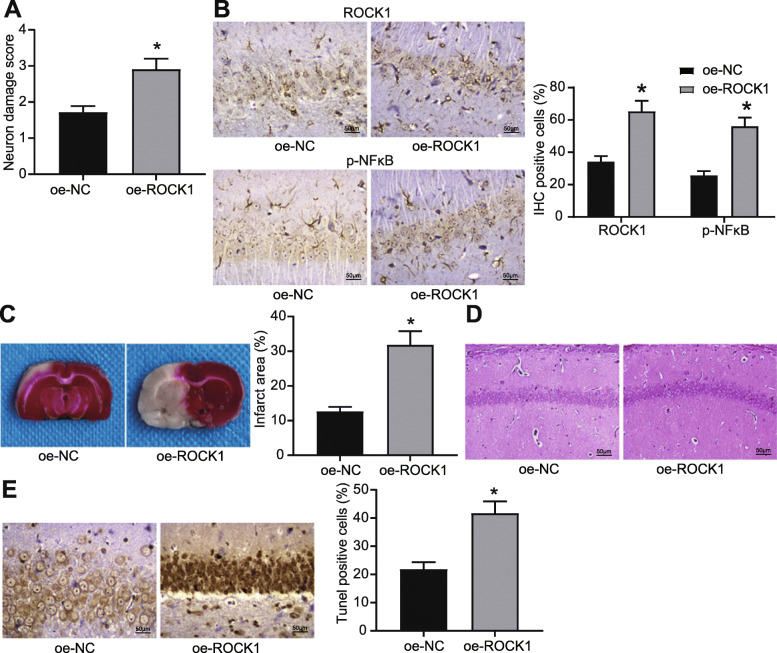
Overexpression of ROCK1 aggravates CIRI in MCAO rats by increasing the extent of NF-κB phosphorylation. **A** Neurological deficit scores for MCAO rats injected with oe-ROCK1. **B** ROCK1 expression and extent of NF-κB phosphorylation in rat hippocampal neuronal cells determined by immunohistochemical staining. **C** Infarction area of rats injected with oe-ROCK1 observed by TTC staining. **D** Damage of neurons in hippocampal regions determined by HE staining. **E** Apoptosis rate of neuronal cells in hippocampal regions examined by TUNEL staining. For panel A, C, and E, comparisons were made using unpaired *t* test. * *p* < 0.05 compared with rats injected with oe-NC. For panel B, comparison was made using two-way ANOVA. * *p* < 0.05 compared with rats injected with oe-NC

To verify that ROCK1 was involved in the DEX-mediated alleviation in CIRI, rats were injected with oe-ROCK1 half an hour before MCAO operation. At 24 h post-operation, neurological deficits scores of rats were measured, which showed that the neurological deficit scores for rats injected with oe-ROCK1 were higher than those injected with oe-NC (Fig. 5A). Immunohistochemistry results exhibited that overexpression of ROCK1 promoted both ROCK1 expression and extent of NF-κB phosphorylation (Fig. 5B). As TTC staining shown, overexpression of ROCK1 increased infarction area in rats (Fig. 5C), while HE staining presented obvious neuronal injury in hippocampal tissues of rats injected with oe-ROCK1 (Fig. 5D). Results of TUNEL staining suggested that overexpression of ROCK1 induced the apoptosis of hippocampal neurons (Fig. 5E).

## Discussion

MCAO modeling has been widely used in studies on CIRI to imitate the ischemic injury in animals [18–20]. We, therefore, performed MCAO modeling on SD rats to establish a CIRI rat model, aiming to observe the effects of DEX treatment on CIRI and to validate the underlying molecular mechanism. In this study, how DEX-mediated miR-214/ROCK1/NF-κB axis regulated the cerebral infarction area and neuronal cell apoptosis in rats receiving MCAO were explored.

Initially, DEX treatment showed damage-relieving effects on CIRI rats induced by MCAO modeling. Thus far, the protective effect of DEX on tissue injury has been reported in the fields of spinal cord injury, myocardial IRI, as well as acute lung injury [21–23]. A preceding study has demonstrated that in the rat hippocampal neurons, DEX can relieve hypoxia/re-oxygenation injury through suppression of mitochondrial fission and apoptosis [24]. Specifically, DEX plays a neuroprotective role against damage induced by intracerebral hemorrhage in the CA1 region of hippocampus [25]. Our experimental statistics further depicted that DEX treatment reduced cerebral infarction area and suppressed neuronal apoptosis in MCAO-modeled rats. Similarly, post-conditioning of DEX has already been found to confer therapeutic impacts on CIRI by decreasing infarction area [26, 27]. Besides, it has also been observed that DEX relieves neuronal injury in the rat hippocampus through reduction of neuronal cell apoptosis [28]. These references further substantiated our results that DEX has the potency to alleviate CIRI.

Our further analyses revealed that miR-214 expression was elevated by DEX treatment in MCAO rats. Accumulating evidences addressed that miRNAs are significant in terms of disease therapy, and miRNA-based therapy is more ideal in gene silencing due to its lower toxicity [29, 30]. miR-214 is a member belonging to the vertebrate-specific family [31], which is involved in peripheral nerve regeneration [32], neural stem cell proliferation [33], as well as therapy of Huntington’s disease, a neurodegenerative disease [34]. Similar to our study, miR-214 is upregulated by DEX treatment in steroid-induced avascular necrosis of the femoral head in a dose-dependent fashion [35]. However, the impacts of DEX-mediated miR-214 were rarely discussed in CIRI previously. In our study, results of TTC and TUNEL assays fully described that inhibition of miR-214 distinctly weakened the therapeutic effects of DEX on neuronal damage in vivo. To our knowledge, we may be the first one reporting that DEX could upregulate miR-214 during the process of CIRI.

The downstream target gene of miR-214 was subsequently explored. We found that miR-214 targeted ROCK1 and negatively regulated the ROCK1 expression. ROCK1 is one of the factors promoting neuronal loss in MCAO-modeled rats, which increases infarction area [36]. The targeting relationship between miR-214 and ROCK1 has been investigated in osteosarcoma and hepatocellular carcinoma cells [11, 37]. However, few works investigated the role of miR-214/ROCK1 axis in CIRI. In the present study, miR-214 negatively regulated ROCK1 in CIRI through direct binding. ROCK1 has been reported to be targeted by many miRNAs in CIRI. For instance, miR-136-5p bound to ROCK1 in CIRI, and overexpressed miR-136-5p led to a reduced ROCK1 expression [38]. In our next action, ROCK1 was detected to enhance the extent of NF-κB phosphorylation. Depletion of NF-κB p65 protein has been revealed to alleviate inflammatory response in CIRI [39]. In contrast, highly expressed NF-κB boosts apoptosis in oxygen-glucose deprivation and reoxygenation (OGD/R) cell model [40]. ROCK1 is closely associated with NF-κB activity under different conditions, such as hepatocellular carcinoma [41], pulmonary fibrosis [42], and arthritis-induced brain cognitive impairment [43]. Coincidentally, a prior work has mentioned that ROCK1 cooperates with the NF-κB pathway to mediate ischemic stroke [44].

## Conclusion

Collectively, DEX treatment has the potency to attenuate cerebral infarction and suppress apoptosis of neurons in rats with CIRI. Our data suggested that DEX might be a candidate drug to treat CIRI. Additionally, we proposed that miR-214 might play a key role in the protection of DEX against CIRI by associating with ROCK1 and the NF-κB pathway in MCAO-modeled rats. Also, our study highlighted the significance of miR-214 for DEX-based CIRI treatment, which may inspire future works on the effect of overexpressed miR-214 on CIRI therapy. However, more efforts are needed to be paid on the validation of miR-214/ROCK1/NF-κB axis on CIRI in vitro, for instance, by establishing an OGD/R cell model.

## Supplementary Information

**Additional file 1.**

## Data Availability

The datasets used and/or analyzed during the current study available from the corresponding author on reasonable request.

## Abbreviations

ANOVA: Analysis of variance
BSA: Bull serum albumin;
CIRI: Cerebral ischemia/reperfusion injury
Dex: Dexmedetomidine
GAPDH: Glyceraldehyde-3-phosphate dehydrogenase
HE: Hematoxylin-eosin
MCAO: Middle cerebral artery occlusion
miR-214: MicroRNA-214
MT: Mutant type
NC: Negative control
OGD/R: Oxygen-glucose deprivation and reoxygenation
ROCK1: Rho-associated kinase 1
RT-qPCR: Reverse transcription quantitative polymerase chain reaction
TCC: 2,3,5-Triphenyltetrazolium chloride
TUNEL: Terminal deoxynucleotidyl transferase-mediated dUTP-biotin nick end labeling assay
UTR: Untranslated region
WT: Wild type
