# Supplementary figures and images for: Protective effects of dexmedetomidine on cerebral ischemia/reperfusion injury via the microRNA-214/ROCK1/NF-κB axis
Source: BMC Anesthesiol. 2021 Aug 16;21:203. doi: 10.1186/s12871-021-01423-5 (PMC8365892; doi:10.1186/s12871-021-01423-5)

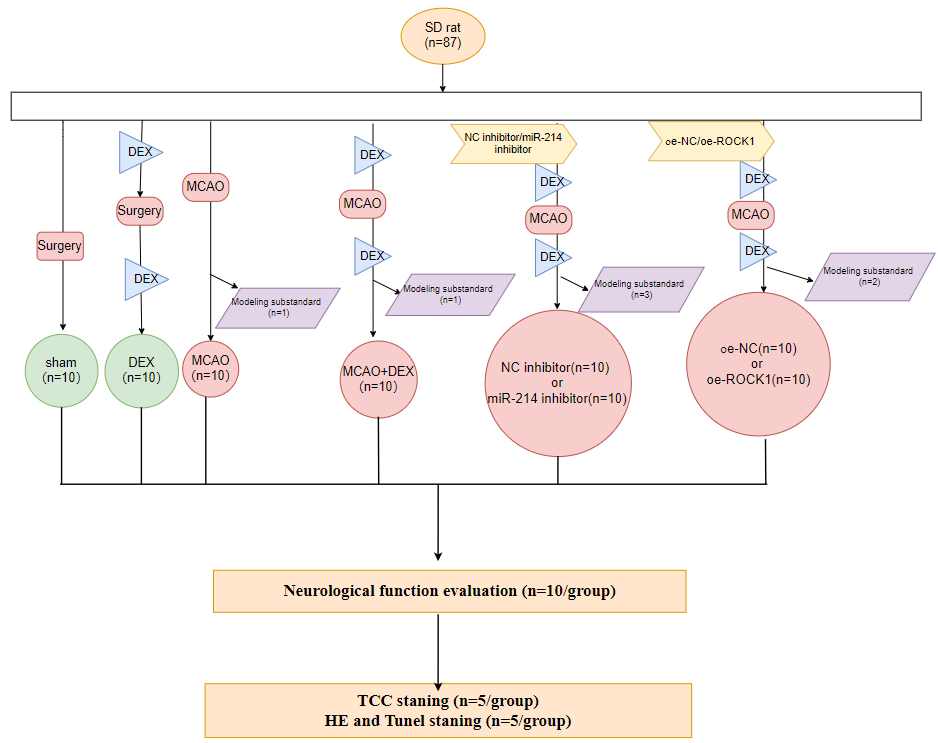


**Supplementary Material** The flow chart

Supplement: Supplementary file 1 — Additional file 1. [file 12871_2021_1423_MOESM1_ESM.docx]
